# The CB_2_ receptor and its role as a regulator of inflammation

**DOI:** 10.1007/s00018-016-2300-4

**Published:** 2016-07-11

**Authors:** Caroline Turcotte, Marie-Renée Blanchet, Michel Laviolette, Nicolas Flamand

**Affiliations:** Centre de recherche de l’Institut universitaire de cardiologie et de pneumologie de Québec, Département de médecine, Faculté de médecine, Université Laval, Quebec, QC G1V 4G5 Canada

**Keywords:** CB_2_ receptor, Cannabinoid, Endocannabinoid, Inflammation, Leukocytes

## Abstract

The CB_2_ receptor is the peripheral receptor for cannabinoids. It is mainly expressed in immune tissues, highlighting the possibility that the endocannabinoid system has an immunomodulatory role. In this respect, the CB_2_ receptor was shown to modulate immune cell functions, both in cellulo and in animal models of inflammatory diseases. In this regard, numerous studies have reported that mice lacking the CB_2_ receptor have an exacerbated inflammatory phenotype. This suggests that therapeutic strategies aiming at modulating CB_2_ signaling could be promising for the treatment of various inflammatory conditions. Herein, we review the pharmacology of the CB_2_ receptor, its expression pattern, and the signaling pathways induced by its activation. We next examine the regulation of immune cell functions by the CB_2_ receptor and the evidence obtained from primary human cells, immortalized cell lines, and animal models of inflammation. Finally, we discuss the possible therapies targeting the CB_2_ receptor and the questions that remain to be addressed to determine whether this receptor could be a potential target to treat inflammatory disease.

## Introduction

The psychotropic effects induced by cannabis promoted its widespread use among the population. These effects are mediated by a cannabinoid receptor that is mainly expressed in the central nervous system, namely CB_1_. The identification of a receptor that is selectively activated by cannabinoids suggested that the human body synthesizes at least one natural ligand for this receptor. This hypothesis was confirmed by the discovery of two high-affinity ligands for the CB_1_ receptor: arachidonoyl-ethanolamide (AEA) [1] and 2-arachidonoyl-glycerol (2-AG) [2]. As these novel lipid mediators were uncovered, a second cannabinoid receptor (CB_2_) was being cloned and characterized. Its expression profile among tissues was found to be distinct from that of CB_1_. It was primarily found in immune cells and was initially not detected in the brain, although this was later proven incorrect by several studies. In light of these findings, the CB_2_ receptor was postulated to be responsible for the immunomodulatory effects of cannabinoids and endocannabinoids. In the past two decades, this hypothesis was tested in a wide array of cellular and animal models. This article offers a comprehensive review of the evidence that was gathered in these studies, with a focus on peripheral inflammation. The CB_2_ receptor’s potential as a therapeutic target in inflammatory disease is also discussed.

## Cloning of the CB_2_ receptor

The non-psychoactive effects of cannabinoids were initially believed to be mediated either centrally or through their interaction with non-receptor proteins. Although there are phytocannabinoids that exert non-psychoactive effects without binding to CB_2_ receptor [e.g., cannabidiol (CBD), cannabigerol (CBG)], discovering the latter explained many of the peripheral effects of cannabinoids. Munro et al. cloned the human CB_2_ receptor in 1993 from the promyelocytic leukaemic cell line HL-60 [3]. To achieve this, cells were treated with dimethylformamide to induce granulocyte differentiation, a cDNA library was prepared, polymerase chain reaction (PCR) was performed using degenerated primers, and the amplification products were cloned and sequenced. One of the clones showed homology to the G-protein-coupled-receptor (GPCR) family and was related to the CB_1_ receptor. The protein encoded by this sequence was found to have 44 % homology with the CB_1_ receptor. This homology increased to 68 % when only the transmembrane portion was considered. Binding assays showed that this receptor had high affinity for the cannabinoid receptor ligands WIN 55,212-2 and CP 55,940, as well as the endocannabinoid AEA and the phytocannabinoid ∆^9^-THC. The authors suggested that the previously described central receptor be named CB_1_ and that this novel, peripheral receptor be named CB_2_.

A few years later, Shire et al. [4] cloned the murine CB_2_ receptor from a mouse splenocyte cDNA library. They found it to be 82 % homologous to the human CB_2_ receptor and to have similar affinity for the ligands AEA, CP 55,940, and ∆^9^-THC. WIN 55,212-2, however, bound the mouse CB_2_ receptor with an affinity six-fold lower than that documented for human CB_2_. This was followed by the cloning of the rat CB_2_ receptor by Brown et al. [5]. The authors also compared the sequence of their clone with those of the mouse and human CB_2_ receptor and found significant differences in protein length, although these were mainly the consequence of disparities in carboxyl termini. Amino acid conservation was highest in the transmembrane regions of the three receptors.

In addition to binding the endocannabinoids AEA and 2-AG, the CB_2_ receptor binds many phytocannabinoids. The pharmacology of endocannabinoids and that of the CB_2_ receptor were rigorously reviewed in the past [6, 7]. Table 1 provides a summary of the various endocannabinoids and phytocannabinoids and their affinity for the human CB_2_ receptor.

**Table 1:** Binding of endocannabinoids and phytocannabinoids to the human CB_2_ receptor

	*K*_i_ (nM)	Model	References
Endocannabinoid			
AEA	371	CHO cells	[8]
	1940	AtT-20 cells	[9]
	795	Sf9 cells	[10]
	3500	CHO cells	[10]
2-AG	949	Sf9 cells	[10]
	650	CHO cells	[10]
Dihomo-γ-LEA	857	AtT-20 cells	[9]
Oleamide	>100,000	HEK-293 cells	[11]
NADA	12,000^b^	Rat spleen	[12]
2-AG-ether	>3000^a^	COS-7 cells	[13]
Phytocannabinoid			
∆^9^-THC	34.6	CHO cells	[8]
∆^8^-THC	39.3	Mouse spleen	[14]
CBN	96.3	CHO cells	[8]
	301	AtT-20 cells	[9]
CBD	2680	CHO cells	[8]
β-Caryophyllene	155	HEK293 cells	[15]

*K*_i_ values were obtained in function of [^3^H]CP 55,940 displacement unless indicated otherwise

*NADA* *N*-arachidonoyl-dopamine, *CBN* cannabinol

^a^[^3^H]HU-243

^b^[^3^H]WIN55212-2

## Available tools to study CB_2_ receptor functions

### Pharmacological compounds

Synthetic cannabinoids, such as CP 55,940 and WIN 55,212-2, were already available when the CB_2_ receptor was cloned. They were subsequently shown to be potent CB_2_ ligands, but also to lack selectivity, as they activate CB_1_ with comparable efficiency. In this respect, several agonists and antagonists were rapidly developed and made available to the scientific community. The most widely used compounds are the agonist JWH 133, and the antagonists SR144528 and AM630. Still, many compounds display good potency and selectivity towards CB_2_. Table 2 contains a comprehensive list of those compounds, as well as their binding potency towards human CB_2_, and in some cases, the other receptors they target.

**Table 2:** CB_2_ agonists and antagonists

	*K*_i_ (nM)	Other targets	References
Agonist			
AM 1241	3.4	TRPA1	[16, 17]
JWH 133	3.4	TRPV1	[18, 19]
GW 405833	3.6–3.92	–	[20]
JWH 015	13.8	–	[8]
HU 308	22.7	–	[21]
L-759,633	6.4	–	[22]
L-759,656	11.8	–	[22]
SER 601	6.3	–	[23]
GP 1a	0.037	–	[24]
GP 2a	7.6	–	[24]
CB 65	3.3	–	[25]
HU 210	0.061–0.52	CB_1_, GPR55, 5-HT_2_	[9, 26, 27]
CP 55,940	0.6–5.0	CB_1_, GPR55	[26, 28]
WIN 55, 212-2	62.3	CB_1_,TRPA1	[9, 17, 28]
Antagonist			
SR144528	0.6–4.1	–	[22, 29]
AM 630	5.6–31.2	TRPA1	[22, 30]
JTE907	35.9	–	[31]

– This compound is not known to activate other receptors besides CB_2_

*TRP* transient receptor potential ion channel

### Knockout mice

The first CB_2_ receptor-deficient mouse was generated by Buckley et al. in 2000 [32]. The *CNR2* gene was inactivated by homologous recombination, by replacing a 341 bp fragment of its coding sequence with the neomycin gene. This mutation eliminated part of intracellular loop 3, transmembrane domains 6 and 7, and the carboxyl extremity of the receptor. Autoradiography experiments confirmed the absence of specific binding of [^3^H]CP 55,940 in the spleen of *CB*_*2*_^−*/*−^ mice. No significant difference in the binding of [^3^H]CP 55,940 between wild-type and knockout animals was found in the brain, supporting that CB_1_-receptor expression was not altered in *CB2* ^−*/*−^ animals. The authors confirmed this by demonstrating that knockout mice were as responsive to the psychotropic effects of Δ^9^-THC as wild-type animals.

*CB*_*2*_^−*/*−^ mice display no morphological differences when compared to their wild-type counterparts. They are normal size and weight, are fertile, have normal litter sizes and care for their young. However, subsequent studies by other groups show that *CB*_*2*_^−*/*−^ mice develop differences at the cellular level. In this regard, Ofeck et al. have demonstrated that *CB*_*2*_^−*/*−^ mice have lower counts of osteoblast precursors and increased numbers and activity of osteoclasts [33]. In consequence, these mice have a low bone mass phenotype that worsens with age. They also present abnormalities in the development of several T and B cell subsets [34]. While this might impair immune homeostasis, *CB*_*2*_^−*/*−^ mice fail to spontaneously develop any observable immune disease. Therefore, they are suitable to study CB_2_ function and have, since, become invaluable tools in cannabinoid research. In this respect, they have been used to define the impact of CB_2_ deficiency in a variety of inflammatory disease models, and the results of these studies will be discussed in the section entitled CB_2_ activation by endocannabinoids in vivo

### Antibodies

As it is the case with numerous GPCRs, CB_2_ protein detection is difficult due to the lack of specificity of primary antibodies. This concept was underscored in a recent study by Marchalant et al. [35], who showed that a commercially available and widely used CB_2_ polyclonal antibody is heavily cross-reactive towards other proteins. Noteworthy, they demonstrated that some of the proteins detected by the antibody were not membrane-bound, ruling out the previously suggested hypothesis that the additional bands represent glycosylation variants of the CB_2_ receptor. Moreover, Graham et al. [36] compared several CB_2_ primary antibodies in flow cytometry experiments on human primary leukocytes. The antibodies which they compared generated different expression patterns between cell types. Therefore, data regarding CB_2_ protein detection must be interpreted with caution.

The detection of the CB_2_ receptor using antibodies can be substituted, to some extent, by the alternate methods. For example, Schmöle et al. [37], recently, generated a bacterial artificial chromosome (BAC) transgenic mouse model that expresses a green fluorescent protein (GFP) under the CB_2_ promoter. This mouse can be used to determine CB_2_ expression in mouse tissues in vitro and in situ, by several techniques, including RT-PCR, qPCR, immunoblot, flow cytometry, and immunofluorescence. This system, based on GFP detection, is an alternative to the use of CB_2_ antibodies on mouse tissues. It is more reliable in the sense that most antibodies directed against GFP are specific and yield reproducible data. However, this kind of approach cannot be used for CB_2_ detection in human primary cells and tissues, which remain problematic. A different strategy that was evaluated by Petrov et al. involves the synthesis of fluorescent CB_2_ agonists [38]. The synthesized compound showed marked selectivity for CB_2_ over the CB_1_, 5-HT_2A_, and 5-HT_2C_ receptors. This agonist was validated as a flow cytometry probe to detect the CB_2_ receptor in cells, and also to evaluate CB_2_-receptor binding using fluorescence microscopy. Other methods of detection could also be added to CB_2_ ligands to use them as probes, such as biotinylation [39].

## CB_2_ expression profiles in human and animal tissues

### Expression profile of CB_2_ among tissues

Upon cloning the human CB_2_ receptor from HL-60 cells, Munro et al. isolated a portion of a rat homologue by PCR [3]. They used this homologue to probe various rat tissues and detected high CB_2_ receptor mRNA levels in the spleen, but not in the liver, nasal epithelium, thymus, brain, lung, or kidney. Cell sorting allowed the authors to associate CB_2_ receptor expression to the monocyte/macrophage population of the spleen rather than T cells. Two years later, Galiègue et al. published the first study describing CB_2_ receptor expression in various human tissues and isolated leukocyte populations [40]. The authors found high CB_2_ mRNA levels in tonsils, spleen, PBMC, and thymus, and were able to detect the CB_2_ protein in tonsils by immunohistochemistry using an anti-CB_2_ polyclonal antibody. They also evaluated CB_2_ receptor mRNA expression in numerous human organs and found it to be absent from most non-immune tissues, with the exception of pancreas, lung, and uterus, which had relatively low mRNA levels. Several reports have, since, shown that the CB_2_ receptor is expressed in both male [41] and female [42, 43] reproductive tissues. In this regard, the CB_2_ receptor exerts an important role in the fertility of both sexes, which has already been extensively reviewed [44–47].

The pattern of CB_2_ receptor expression among human tissues is consistent between studies. More groups have reported the presence of the CB_2_ receptor mRNA and protein in the human spleen [48] and tonsils [49]. Moreover, the high level of CB_2_ expression in human immune tissues was also reported in murine and rodent spleen [37, 50–56] and thymus [37, 54].

The presence and role of the CB_2_ receptor in the central nervous system have yet to be fully elucidated, and the issue was discussed in a review article recently published by Atwood and Mackie [57]. It was initially believed that it was not expressed in non-immune cells of the central nervous system, because Munro et al. did not detect CB_2_ receptor mRNA in any brain part when they cloned the receptor [3], which is supported by many studies [40, 54, 58, 59]. However, we now know that the CB_2_ receptor is not completely absent from the brain, since it is expressed in microglia [60]. Still, the concept of the CB_2_ receptor being a second central cannabinoid receptor is up to debate for three main reasons: (1) a study showed that the CB_2_ receptor agonists JWH-015 and JWH-133 modulate peripheral neuron functions [61] and (2) the CB_2_ receptor was detected in the uninjured brain by immunochemistry on numerous occasions [62–64], and (3) a recent study found that hippocampal principal neurons express CB_2_ mRNA, and that CB_2_-selective agonist HU-308 modulated the activity of these cells [65]. Conversely, a study that relied on GFP detection to determine the expression of the CB_2_ receptor in the murine brain showed that the signal is located in microglia [37]. Therefore, the lack of reliability of the antibodies that were used in immunochemistry experiments stresses the need for more research to expand our knowledge on the involvement of the CB_2_ receptor in the central nervous system and neuroinflammation.

In 2009, Liu et al. showed that two distinct isoforms of the CB_2_ receptor exist [66]. The novel CB_2_ isoform was a splicing variant of the earlier cloned receptor, and was identified from a human neuroblastoma cDNA library. Splicing variants were also discovered in mice and rats, although their genomic structures and transcripts were different from those found in humans. Furthermore, the two human variants were found to display tissue-specific expression patterns. While the classical CB_2_ isoform was predominantly found in spleen and other immune tissues, the novel isoform was detected in higher levels in testis and brain regions of the reward system. The identification of this new CB_2_ variant could shed some light on the confusing expression patterns that were previously reported. Finally, it underscores the possibility of a role for CB_2_ in reproductive and central nervous systems that are distinct from the immunomodulatory role of the classical CB_2_ isoform.

### CB_2_ expression in immune cells

It is well known that the CB_2_ receptor is widespread among cells of the immune system. Table 3 provides the literature associated with the expression of the CB_2_ receptor in human leukocytes. Every cell type that has been investigated was found to express both mRNA and protein in at least one report. However, there is conflicting data associated with a few cell types. For example, there is no consensus in the literature regarding the presence of the CB_2_ receptor in human neutrophils. Of note, not every study was conducted on purified, eosinophil-depleted neutrophils. Given that eosinophils have very abundant amounts of CB_2_ receptor mRNA, a small number of eosinophils among the neutrophil sample could result in a false positive. This is consistent with the observation that CB_2_ levels are lower in neutrophils than in eosinophils.

**Table 3:** CB_2_ receptor expression in human leukocytes

Cell types	Data	CB_2_ expression	References
B cells	mRNA	+	[36, 40, 68, 69]
	Protein	+	[49, 68, 70]
Basophils	mRNA	+	[71]
Dendritic cells	mRNA	+	[56]
	Protein	+	[56]
Eosinophils	mRNA	+	[71–74]
	Protein	+	[74]
Mast cells	mRNA	+	[71]
Macrophages	mRNA	+	[75]
	Protein	+	[48, 75, 76]
Microglia	mRNA	+	[60]
	Protein	+	[60]
Monocytes	mRNA	+	[36, 40, 68, 75, 77, 78]
	Protein	+	[68, 75, 78]
NK cells	mRNA	+	[36, 40, 69]
	Protein	+	[49]
Neutrophils	mRNA	+ −	[36, 40, 71] [72–74]
	Protein	+ −	[79] [74]
Platelets	mRNA	+	[71]
	Protein	+ −	[80] [81]
T cells	mRNA	+	[36, 40, 68, 69]
	Protein	+	[49, 68, 82]

As discussed in the previous section, the scientific community should always be critical when interpreting protein data, especially of GPCRs. A large number of researchers have now reported expression data obtained with commercially available antibodies, and most of them relied on a positive control to validate their results. It was later underscored that in the case of the CB_2_ receptor, a reliable negative control is absolutely necessary to confirm that the signal is not generated by non-specific binding of the antibody [35, 67].

## CB_2_ receptor signaling

The CB_2_ receptor was associated to the GPCR family when it was cloned. However, the signal transduction pathways induced by CB_2_ receptor activation are far less characterized than those of CB_1_. CB_1_ is known to inhibit adenylyl cyclase, to modulate ion channels, and to activate numerous downstream signaling events, including p38 and p42/44 MAPK (ERK-1/2), PI3K, calcium mobilization (phospholipase C/IP_3_), the arachidonic acid cascade, and nitric oxide production (reviewed in [83]). A few studies have aimed to compare the signaling events of CB_1_ and CB_2_ in a given cell system and found some divergences between the two receptors. This section recapitulates the evidence regarding the signaling events downstream of the CB_2_ receptor.

### G_i/o_ protein coupling and adenylyl cyclase inhibition

Like the CB_1_, the CB_2_ receptor couples with G_i/o_ proteins. This was established by Slipetz et al. who found that in CB_2_-transfected Chinese Hamster Ovary (CHO) cells, pretreatment with pertussis toxin (PTX) abolished the effect of cannabinoids on forskolin-induced cAMP production [84]. Other groups using CB_2_-transfected cell models found signaling events to be PTX-sensitive, supporting the involvement of G_i/o_ proteins [85, 86]. This interaction was later confirmed in murine microglial cells [87], the murine macrophage cell line J774-1 [88], the human promyelocytic cell line HL-60 [89–91], and human bronchial epithelial cells [92]. Since it has proven to couple to G_i/o_ proteins, the impact of CB_2_ activation on adenylyl cyclase activity was also investigated. As expected, adenylyl cyclase was inhibited upon treatment of cells with CB_2_ receptor agonists and/or synthetic cannabinoids, resulting in a decrease in intracellular cAMP levels [84, 85, 93, 94].

### Potassium channels

As opposed to the CB_1_ receptor, the CB_2_ receptor does not appear to couple to potassium channels. A study by Felder et al. [9] investigated the possible modulation of inwardly rectifying potassium current (*K*_ir_) channels in CB_2_-transfected AtT-20 cells. In these cells, activation of the CB_2_ receptor with WIN 55,212-2 failed to have an impact on *K*_ir_. Another study showed that in *Xenopus laevis* oocytes co-expressing the CB_2_ receptor and G-protein-gated inwardly rectifying potassium (GIRK) channels, WIN 55,212-2 failed to induce consistent coupling of the CB_2_ receptor to GIRK channels [95]. Of note, the CB_1_ receptor was able to couple with GIRK channels and to modulate agonist-induced currents in the same cellular model. This important difference between CB_1_ and CB_2_ receptors established CB_2_ as a functionally distinct receptor.

### Mitogen-activated protein kinases (MAPK)

Signal transduction pathways induced by CB_2_ receptor activation were first investigated in CB_2_-CHO cells by Bouaboula et al. [86]. They found that upon CP 55,940 addition, adenylyl cyclase inhibition was followed by ERK-1/2 phosphorylation. This effect was significantly diminished by the protein kinase C (PKC) inhibitor GF 109203X, suggesting that PKC was involved in MAPK activation. Moreover, they were able to confirm their findings in HL-60 cells, which express the CB_2_ receptor. Another group investigated MAPK activation by various CB_2_ ligands in HL-60 cells and found that CP 55,940, 2-AG, and AEA increased ERK-1/2 phosphorylation [89]. This effect was blocked by the CB_2_ receptor antagonist SR144528 and was stronger in cells stimulated by 2-AG and CP 55,940 than in those treated with AEA. MAPK activation downstream of CB_2_ activation was also demonstrated in vitro in murine osteoblasts [96], in DAUDI leukemia cells [94], murine microglia [97], and human primary monocytes [78]. Finally, this pathway was showed to be activated in vivo, in a mouse model of acute experimental pancreatitis. In this model, a CB_2_ receptor agonist reduced inflammation through the p38-MK2 pathway [98].

### Intracellular calcium concentrations and phospholipase C activity

A study conducted in calf pulmonary endothelial cells showed that CB_2_ activation modulates intracellular calcium concentrations [99]. In this model, AEA initiated phospholipase C (PLC) activation and inositol 1,4,5-triphosphate (IP_3_) production, which led to intracellular Ca^2+^ release from the endoplasmic reticulum, as well as an increase in mitochondrial Ca^2+^. This effect of AEA was not mimicked by arachidonic acid (AA), was blocked by SR144528, and was unchanged by treatment with SR141716A, confirming the involvement of the CB_2_, but not the CB_1_ receptor. Another group later confirmed this in HEK-293 cells co-expressing the CB_2_ receptor with chimeric G_i_ and G_o_ proteins [100]. In this model, treatment with CP 55,940 or other CB receptor agonists was found to increase intracellular Ca^2+^ levels. The phospholipase C inhibitor U73122 abrogated the effect of CP 55,940 on calcium mobilization, as did thapsigargin. This evidence shows that in these cells, CB_2_ receptor activation induces calcium mobilization via the PLC-IP_3_ signaling pathway.

## In vitro studies of CB_2_ receptor functions

### CB_2_ activation by endocannabinoids in vitro

The endocannabinoids 2-AG and AEA both act on various immune cell types through CB_2_ receptor activation (summarized in Table 4). Interestingly, there is a sharp contrast between the anti-inflammatory effects that are triggered by the two lipids. 2-AG was most often found to modulate functions related to leukocyte recruitment, such as chemokine release, adhesion to fibronectin, and migration. This positive regulation of immune cell recruitment by 2-AG is the main pro-inflammatory effect of endocannabinoids or cannabinoids in vitro that has been reported. AEA, on the other hand, was found to downregulate leukocyte functions, such as pro-inflammatory cytokine release and nitric oxide production. A few reports also show increased production of the anti-inflammatory cytokine IL-10 by cells treated with AEA. In all cases, the involvement of the CB_2_ receptor was confirmed by the use of a selective antagonist. However, it is still possible that endocannabinoid metabolites are involved in the reported effects. Noteworthy, this hypothesis was tested in human eosinophils which were shown to migrate in response to 2-AG [101]. In this model, the effect of 2-AG on eosinophil transmigration was blocked by the pre-incubation of cells with a CB_2_ receptor antagonist. However, a CB_2_-selective agonist failed to mimic the impact of 2-AG, and its 15-LO-derived metabolites were suggested to be necessary for eosinophils to migrate. Therefore, the successful blockade of endocannabinoid-induced effects with a CB_2_ antagonist does not always rule out the possibility that other mediators, notably endocannabinoid metabolites, are involved as well [102]. This concept could explain why endocannabinoids can induce both pro- and anti-inflammatory effects.

**Table 4:** CB_2_-mediated effects of endocannabinoids on immune cell functions

Cell type	Species	Endocannabinoid	Effects	References
Anti-inflammatory effects				
Astrocytes	Rat	AEA	↓TNF-α	[103]
Dendritic cells	Human	AEA	↓ IL-6, IL-12 and IFN-α	[104]
Microglia	Mouse (BV-2 cell line)	AEA	↓ Nitric oxide	[105]
	Mouse	AEA	↑ IL-10	[106]
			↑ IL-10 ↓ IL-12p70 and IL-23	[107]
	Rat	AEA	LPS-induced nitric oxide release	[108]
Neutrophils	Human	2-AG	↓ fMLP-induced migration	[79]
Splenocytes	Human	AEA	↓ Primary and secondary antibody formation	[109]
T cells (not separated)	Human	AEA	↓ Cell proliferation	[110]
		2-AG	↓ SDF-1-induced migration	[111]
CD4+ T cells	Human	AEA	↓ IL-17, IFN-γ and TNF-α	[110]
CD8 + T cells	Human	AEA	↓ IFN-γ and TNF-α	[110]
	Human	AEA	↓ SDF-1-induced migration	[112]
Pro-inflammatory effects				
B cells	Human	2-AG	↑ Migration	[113]
	Mouse	2-AG	↑ Migration	[114, 115]
Dendritic cells	Human	2-AG	↑ Migration	[116]
Eosinophils	Human	2-AG	↑ Migration	[74, 117]
	Human	2-AG	↑ Migration ↑ LTC_4_ and EXC_4_ synthesis	[101]
Macrophages	Mouse (peritoneal)	2-AG	↑ Zymosan phagocytosis	[118]
	Human (HL-60)	2-AG	↑ Actin polymerization ↑ Adhesion to fibronectin	[119]
			↑ MCP-1 and IL-8	[120]
Microglia	Mouse (BV-2 cell line)	2-AG	↑ Migration	[121]
Monocytes	Human	2-AG	↑ Adhesion to fibronectin	[122]
			↑ Migration	[119]
NK cells	Human	2-AG	↑ Migration	[123]
T cells	Human (Jurkat)	2-AG	↑ L- and P-selectin ↑ Adhesion and transmigration	[124]

*TNF* tumor necrosis factor, *IL* interleukin, *IFN* interferon, *LPS* lipopolysaccharide, *fMLP* formyl-Met-Leu-Phe, *SDF* stromal cell-derived factor, *LTC*_*4*_ leukotriene C_4_, *EXC*_*4*_ eoxin C_4_, *MCP* monocyte chemoattractant protein

### CB_2_ activation by exogenous agonists in vitro

In contrast to endocannabinoids, CB_2_ receptor agonists have only been shown to exert anti-inflammatory effects on leukocytes, which are detailed in Table 5. Some of the studies were performed using a non-selective cannabinoid, but the involvement of the CB_2_ receptor was always confirmed with an antagonist. In addition to downregulating leukocyte functions, such as cytokine release, reactive oxygen species production and migration, CB_2_ agonists limited HIV-1 expression, and replication in human macrophages and microglia [75, 125].

**Table 5:** Effects of CB_2_ agonists on immune cell functions

Cell type	Species	Agonist	Effects	References
Astrocytes	Human	WIN 55,212-2	↓ Nitric oxide ↓ TNF-α, IL-10, MCP-1 and CCL5	[126]
Dendritic cells	Mouse	∆^9^-THC	↑ NF-κB-dependent apoptosis	[127]
		GP1a	↓ MMP-9 ↓ Migration	[128]
Monocytes	Human	JWH-015	↓ CCL2 and CCL3-induced migration	[78]
		HU-308 JWH-133	↓ TNF-α-induced transendothelial migration	[129]
Macrophages	Human (monocyte-derived)	JWH-133	↓ Expression of 35 genes upregulated by LPS	[130]
		JWH-133 GP1a O-1966	↓ HIV-1 replication	[75]
	Mouse (RAW264.7)	WIN 55,212-2	↓ Reactive oxygen species	[131]
			↓ Nitric oxide	[132]
	Mouse (peritoneal)	∆^9^-THC	↓ RANTES-induced migration	[133]
		JWH-133	↑ IL-10 ↓ IL-12p40	[134]
	Mouse (clone 63)	∆^9^-THC	↓ Activation of CD4+ T cells	[59]
Mast cells	Rat (RBL-2H3)	WIN 55,212-2 CP 55,940	↓ β-Hexosaminidase release	[135]
Microglia	Human	WIN 55,212-2	↓ HIV-1 expression	[125]
	Rat	JWH-015	↓ LPS-induced TNF-α production ↓ Migration	[136]
Neutrophils	Mouse	JWH-133	↓ MIP-2α-induced migration	[137]
	Human	JWH-133	↓ TNF-α-induced MMP-9 release	[138]
Splenocytes	Human	∆^9^-THC	↓ Primary and secondary antibody formation	[109]
T cells	Human	∆^9^-THC	↓ Th2 cytokine production	[139]
	Human (Jurkat)	CP 55,940 WIN 55,212-2 JWH-015	↓ SDF-1-induced migration	[140]
	Mouse	O-1966	↓ NF-κB activation ↑ SOCS5 expression ↑ IL-10	[141]
CD8+ T cells	Human	JWH-015	↓ SDF-1-induced migration	[140]

*CCL* chemokine (C–C motif) ligand, *NF-*κ*B* nuclear factor kappa-light-chain-enhancer of activated B cells, *MMP* matrix metallopeptidase, *MIP* macrophage inflammatory protein, *HIV* human immunodeficiency virus, *SOCS* suppressor of cytokine signaling

## In vivo studies of CB_2_ receptor functions

### Impact of CB_2_ knockout in inflammation models

Transgenic mice have greatly contributed to our understanding of this receptor’s role in human disease, including inflammatory conditions. In this regard, several models have shown that mice that are lacking the CB_2_ receptor have exacerbated inflammation (summarized in Table 6). The effects that were usually observed in *CB*_*2*_^−*/*−^ animals included increased leukocyte recruitment (often neutrophils) and pro-inflammatory cytokine production, which often caused tissue damage. Conversely, one study found CB_2_-deficient mice to be in better condition than the wild-type group [142]. However, the model was cecal ligation-induced sepsis, a condition in which efficient bacterial clearance by the immune system is vital. The authors’ observations that the *CB*_*2*_^−*/*−^ group had less mortality and less bacterial invasion was explained by the lower levels of IL-10 in these mice, which might have led to a better phagocytic response. Overall, these findings are consistent with the other reports of increased immune cell functions in the absence of the CB_2_ receptor.

**Table 6:** Anti-inflammatory effects of CB_2_ receptor deletion in inflammation models

Model	Species	Genotype	Effects	References
DNFB-induced hypersensitivity	Mouse	*CB*_*2*_^−*/*−^	↑ Neutrophil recruitment ↑ Ear swelling	[143]
Hepatic ischemia–reperfusion injury	Mouse	*CB*_*2*_^−*/*−^	↑ Neutrophil recruitment ↑ Inflammatory cytokines ↑ Liver damage	[144]
TNBS-induced colitis	Mouse	*CB*_*2*_^−*/*−^	↑ Colitis ↑ TNF-α and IL-1β	[145]
Myocardial ischemia–reperfusion injury	Mouse	*CB*_*2*_^−*/*−^	↑ Neutrophil and macrophage infiltration ↓ IL-10	[146]
Traumatic brain injury	Mouse	*CB*_*2*_^−*/*−^	↑ TNF-α, iNOS and ICAM mRNA ↑ Blood–brain barrier permeability	[147]
Cecal ligation-induced sepsis	Mouse	*CB*_*2*_^−*/*−^	↓ IL-10 ↓ Bacterial invasion ↓ Mortality	[142]

*DNFB* 2,4-dinitrofluorobenzene, *TNBS* trinitrobenzenesulfonic acid, *iNOS* inducible nitric oxide synthase, *ICAM* intercellular adhesion molecule

### CB_2_ activation by exogenous agonists in vivo

The potential of activating CB_2_ in vivo to treat inflammation has been investigated in numerous studies. Two main strategies are employed: (1) the administration of a CB_2_ receptor agonist; and (2) the administration of an endocannabinoid hydrolysis inhibitor to augment endocannabinoid signaling.

The administration of CB_2_ receptor agonists has been performed in several inflammation models. Table 7 summarizes the data that were generated with this approach. In many instances, the chosen agonist was not CB_2_-selective and targeted both cannabinoid receptors, in which case, the involvement of CB_2_ was confirmed by showing that the treatment of animals with a CB_2_ antagonist abrogated the effects of the cannabinoid receptor agonist. Altogether, the results of those studies point to the conclusion that CB_2_ activation improves inflammation in mice. The recruitment of leukocytes to tissues and the production of pro-inflammatory cytokines and reactive oxygen species were downregulated in various inflammation models. In the case of atherosclerosis, two studies showed not only a decrease in inflammatory cells and mediators upon cannabinoid treatment, but also a slower progression of the disease [148, 149]. Indeed, oral ∆^9^-THC administration, at doses that are suboptimal for inducing psychotropic effects, resulted in reduced atherosclerotic lesion development. Since these effects of ∆^9^-THC were shown to be mediated by the CB_2_ receptor, this supports that a selective CB_2_ receptor agonist might be a valuable tool for the treatment of atherosclerosis.

**Table 7:** Anti-inflammatory effects of CB_2_ agonists in animal models of inflammation

Model	Species	Treatment	Effects	References
Atherosclerosis	Mouse	∆^9^-THC	↓Atherosclerotic lesions ↓ Macrophage infiltration ↓ Leukocyte adhesion	[149]
		WIN 55,212-2	↓Atherosclerotic lesions ↓ Macrophage infiltration ↓ MCP-1, IL-6 and TNF-α	[148]
Breast cancer cell injection	Mouse	∆^9^-THC	↓ Splenocyte proliferation	[150]
Brain ischemia	Mouse	JWH-133	↓ Microglia and macrophage infiltration ↓ IL-6, MCP-1, MIP-1α, CCL-5 and TNF-α ↓ iNOS	[151]
Experimental autoimmune encephalomyelitis	Mouse	∆^9^-THC JWH-133	↓ Monocyte recruitment ↓ IFN-γ and IL-2 ↓ T cell proliferation	[152]
Hepatic ischemia–reperfusion injury	Mouse	∆^8^-THCV	↓ Hepatic injury ↓ CCL3, CXCL2 and TNF-α ↓ Neutrophil infiltration	[153]
Germinal matrix hemorrhage-induced neuroinflammation	Rat	JWH-133	↓ TNF-α ↓ Microglia accumulation	[154]
*L. pneumophila* infection	Mouse	∆^9^-THC	↓ IFN-γ and IL-12	[155]
Influenza virus infection	Mouse	∆^9^-THC	↓ Lymphocyte and monocyte recruitment ↓ Viral hemagglutinin	[156]
Myocardial ischemia–reperfusion injury	Mouse	WIN 55,212-2	↓Myeloperoxidase ↓ IL-1β and IL-8	[157]
Ovalbumin-induced asthma	Guinea pig	CP 55,940	↓Myeloperoxidase ↓ Mast cell degranulation ↓ TNF-α and PGD_2_	[158]
LPS-induced interstitial cystitis	Mouse	JWH-015	↓ Leukocyte infiltration ↓Myeloperoxidase ↓ TNF-α, IL-1α and IL-1β	[159]
Sepsis	Mouse	HU308	↓ Adherent leukocytes in submucosal venules	[160]
Spinal cord injury	Mouse	O-1966	↓ Leukocyte infiltration ↓ CXCL9 and CXCL11 ↓ IL-23p19 and IL-23R ↓ TLR expression	[161]
Stress-induced neuroinflammation	Mouse	JWH-133	↓ TNF-α and MCP-1 ↓ COX-2, iNOS and NF-κB	[162]
Traumatic brain injury	Mouse	O-1966	↓ Microglia and macrophage infiltration ↓ Blood–brain barrier disruption	[163]

*PGD*_*2*_ prostaglandin D_2_, *COX-2* cyclooxygenase-2

### CB_2_ activation by endocannabinoids in vivo

The most widely used approach to investigate the impact of endocannabinoids in vivo is the blockade of their hydrolysis, as it is an efficient way to increase their levels in tissues. Despite the numerous studies that have used this method in animal models, it is still unclear whether the effects of endocannabinoids are pro- or anti-inflammatory. This is due, in part, to the presence of numerous enzymes that can metabolize them into other bioactive lipids. The main pathway is hydrolysis into AA by lipases, such as MAG lipase for 2-AG [164] and FAAH for AEA [165]. AA is a precursor for the biosynthesis of leukotrienes, prostaglandins, and other lipid mediators of inflammation. Alternatively, endocannabinoids can undergo oxidation and the biological effects of the metabolites that originate from these pathways are not very well characterized [166]. Therefore, it is not possible to conclude that endocannabinoids exert their effects through CB_2_ in an inflammation model unless this is confirmed by the genetic or pharmacological blockade of the receptor. In this respect, Table 8 only presents studies that have thoroughly confirmed the involvement of the CB_2_ receptor in the effects they observed.

**Table 8:** Anti-inflammatory effects of CB_2_ activation by endocannabinoids in mouse models of inflammation

Model	Treatment	Effects	References
ConA-induced hepatitis	AEA	↓ Inflammatory cytokines	[144]
Carrageenan-induced acute inflammation	URB602	↓ Edema ↓ Nociception	[167]
Experimental autoimmune encephalomyelitis	WWL70	↓ iNOS, COX-2, TNF-α and IL-1β ↓ T cell infiltration ↓ Microglial activation ↓ NF-κB activation	[168]
LPS-induced acute lung injury	JZL184	↓ Leukocyte infiltration ↓ BALF cytokines and chemokines	[169]
LPS-induced inflammatory pain	FAAH KO	↓ Edema ↓ TNF-α and IL-1β	[170]
	FAAH KO, PF-3845, URB597 or OL-135	↓ Allodynia	[171]
Kaolin and carrageenan-induced osteoarthritis	URB597	↓ Leukocyte rolling ↓ Microvascular perfusion	[172]
TNBS-induced colitis	JZL184	↓ Submucosa edema ↓ Leukocyte infiltration ↓ Mucosal IL-6 and IL-1β ↓ Circulating inflammatory markers	[173]

*ConA* concanavalin A, *BALF* bronchoalveolar lavage fluid

A limited number of studies reported pro-inflammatory effects of endocannabinoids in vivo, and only three of those (listed in Table 9) were confirmed to involve the CB_2_ receptor. In two models of dermatitis in mice, treatment with the CB_2_ antagonist SR144528 improved inflammation by inhibiting granulocyte recruitment and pro-inflammatory mediator production [174, 175]. In both cases, this translated in a measurable decrease in swelling. As presented above in Table 6, 2-AG has been implicated in the recruitment and migration of B and T cells, dendritic cells, eosinophils, monocytes, and natural killer cells in a CB_2_-dependent manner, which could very well translate to in vivo studies. However, to this day, there is no published data demonstrating that exogenous cannabinoids and selective CB_2_ receptor agonists have pro-inflammatory effects. Therefore, it is possible that the pro-inflammatory effects of endocannabinoids that are presented in Table 9 are a result of CB_2_ activation and/or the action of one or more endocannabinoid metabolites [102].

**Table 9:** Pro-inflammatory effects of CB_2_ signaling in mouse models of inflammation

Model	Treatment	Effects	References
Primary immunization	2-AG	↑ Delayed-type hypersensitivity ↑ DC migration to draining lymph nodes	[116]
TPA-induced ear inflammation	SR144528	↓ Neutrophil recruitment ↓ Swelling ↓ LTB_4_ synthesis	[179]
Oxazolone-induced dermatitis	SR144528	↓ Eosinophil recruitment ↓ Swelling ↓ MCP-1, MIP-1 and TNF-α	[175]

*TPA* 12-*O*-tetradecanoylphorbol-13-acetate

Of note, many disorders cause a change in CB_2_ receptor protein levels, due to pre-existing pro-inflammatory conditions. In multiple sclerosis and amyotrophic lateral sclerosis, for instance, the expression of CB_2_ in microglia is increased, both in human tissues and mouse models [176, 177]. A similar effect was reported in a rodent model of neuropathic pain [178]. This certainly facilitates the impact of CB_2_ receptor activation by exogenous agonists of endocannabinoids in these inflammation models.

## The CB_2_ receptor as a potential therapeutic target

While there is a large body of evidence supporting that CB_2_ receptor activation has anti-inflammatory effects, it has yet to be targeted to treat human disease. In the two previous sections, we presented in vitro and in vivo studies that suggested a role for the CB_2_ receptor in numerous inflammatory conditions. In this section, we discuss the potential of the CB_2_ receptor as a target in the treatment of chronic inflammatory diseases, such as rheumatoid arthritis, atherosclerosis, and inflammatory bowel disease.

### Potential in rheumatoid arthritis

Rheumatoid arthritis (RA) is an inflammatory disease that affects approximately 1 % of the adult population worldwide. RA is characterized by chronic inflammation of the synovium, cartilage destruction, and bone loss. Patients with RA exhibit an influx of innate (neutrophils, macrophages) and adaptive (lymphocytes) immune cells in the synovial cavity. These cells promote inflammation and connective tissue damage by producing cytokines (TNF-α, IL-6, IL-1β), pro-inflammatory lipids, and metalloproteinases (MMPs). The synovial lining becomes hyperplastic and an invasive structure (the pannus) is formed. Osteoclasts become exaggeratedly activated and cause bone resorption [180].

2-AG and AEA are present in the synovial fluid of patients with RA, but not healthy volunteers, suggesting an involvement of the endocannabinoid system in the disease. CB_1_ and CB_2_ mRNA and proteins were also found in the synovial tissues of RA patients [181]. CB_2_ activation can inhibit the production of pro-inflammatory cytokines and MMP release from fibroblast-like synoviocytes (FLSs) [182, 183]. It can also promote osteoblast differentiation in vitro [33, 184] and inhibit FLS proliferation [182]. These observations indicate that CB_2_ receptor activation in RA joints could improve multiple aspects of the disease, including inflammation, FLS hyperplasia, and bone loss.

In vivo, CB_2_ agonists have proven to be beneficial in a murine model of rheumatoid arthritis, collagen-induced arthritis (CIA). One study showed treatment with the CB_2_ receptor agonist JWH 133 to improve arthritis severity and to reduce bone destruction and leukocyte infiltration in the joints [183]. Another group investigated the impact of a different CB_2_-selective agonist, HU-308. They found that the agonist decreased swelling, synovial inflammation, and joint destruction, in addition to lowering circulating antibodies against collagen II [185]. Finally, the agonist HU-320 ameliorated established CIA [186]. Of note, CB_2_ agonists did not prevent the onset of RA in any of those reports, as there were no differences in disease incidence between groups.

This growing body of evidence establishes the CB_2_ receptor as a promising target for the treatment of RA. In all three of the above-mentioned studies, the CIA model was used to test CB_2_ agonists. Given that there is no animal model of RA that perfectly duplicates all aspects the human condition, these findings should be confirmed in different models.

### Potential in atherosclerosis

Atherosclerosis is an inflammatory disease that is characterized by the presence of arterial plaques. These lesions contain immune cells, lipid-laden macrophages (foam cells), cholesterol, smooth muscle cells, and collagen fibres [187]. The physical rupture of the plaques causes the occlusion of arteries, which can lead to tissue infarction. Plaque development is influenced by inflammatory mediators, such as cytokines and chemokines, which are crucial to the recruitment of immune cells to the intima. In this respect, therapies that would downregulate the production of these mediators could reduce the progression of atherosclerotic lesion development. Since the CB_2_ receptor is known to decrease the production of numerous chemokines and to inhibit leukocyte migration in vitro and in vivo, it emerged as a potential target to treat atherosclerosis.

A recent study specifically aimed to characterize the endocannabinoid system in human foam cells [188]. The authors found that the CB_2_ agonist JHW-015 significantly decreased oxLDL accumulation in these macrophages. Moreover, it reduced the production of TNF-α, IL-6, and IL-10 and the expression of CD36, a scavenger receptor that is responsible for the uptake of modified lipoproteins by macrophages and the induction of foam cell formation. The endocannabinoids 2-AG and AEA mimicked these effects, which were block by the CB_2_ antagonist SR144528. These findings are in accordance with a previous study which showed that CB_2_ activation by WIN 55,212-2 reduces the oxLDL-induced inflammatory response in rat macrophages [131].

As briefly discussed in the section entitled In vivo studies of CB_2_ receptor functions, the role of the CB_2_ receptor was investigated in mouse models of atherosclerosis. The first study to demonstrate the benefits of CB_2_ activation in atherosclerosis was performed in *ApoE* ^−*/*−^ mice using low doses of the cannabinoid ∆^9^-THC, which diminished inflammation and blocked the progression of the disease [149]. These effects were prevented by SR144528, confirming the involvement of the CB_2_ receptor. The anti-atherosclerotic effects of CB_2_ in the *ApoE* ^−*/*−^ model were later confirmed with WIN 55,212-2 as an agonist, and the antagonist AM630 confirmed the mechanism to be CB_2_-dependent [148, 189]. In *Ldlr* ^−*/*−^ *CB*_*2*_^−*/*−^ double knockout mice, lesional macrophage and smooth muscle cell contents were higher than in *Ldlr* ^−*/*−^ *CB*_*2*_^+*/*+^ animals [190]. In *Ldlr* ^−*/*−^ mice deficient for CB_2_ in hematopoietic cells only, plaque area after 12 weeks on an atherogenic diet was larger than in mice with no CB_2_ deficiency [191].

In summary, a large body of evidence strongly suggests that CB_2_ receptor activation is an appropriate target for atherosclerosis treatment. CB_2_ agonists have the potential to be beneficial on many levels, as they were shown to improve inflammatory cell recruitment and activation, lipid uptake by macrophages, and the size of atherosclerotic plaques. However, a few reports show conflicting data, especially in the *Ldlr* ^−*/*−^ model. A report shows unaltered lesion size following WIN 55,212-2 treatment in this model, although CB_2_ receptor activation did decrease lesional macrophage accumulation [192]. Another group treated *Ldlr* ^−*/*−^ mice with JWH-133 and found no significant effect on lesion size or on their content in macrophages, lipids, smooth muscle cells, collagen, and T cells [193]. More investigation is required to determine the causes of these discrepancies before moving forward in the development of therapies targeting CB_2_ for atherosclerosis.

### Potential in inflammatory bowel disease

Inflammatory bowel disease (IBD) includes two main conditions: ulcerative colitis and Crohn’s disease. They are caused by an excessive immune response and can affect any part of the gastrointestinal tract [194]. The endocannabinoid system first gained interest in IBD pathophysiology in light of a study that described a protective effect of CB_1_ in DNBS-induced colitis [195]. Cannabinoids were then shown to enhance epithelial wound healing in a CB_1_-dependent fashion [76]. The authors of the latter study also evaluated the expression of cannabinoid receptors in human IBD tissue by immunochemistry. They found that the CB_1_ receptor was expressed in the normal human colon, but that CB_2_ expression was higher in IBD tissues and that its presence was concentrated in plasma cells and macrophages. These findings raised the hypothesis that the CB_2_ receptor was also involved in the inflammatory component of IBD.

A subsequent study reported that a FAAH inhibitor decreased inflammation in the TNBS-induced colitis model, and that the deletion of either CB_1_ or CB_2_ abrogated this effect [196]. In the same colitis model, the use of the MAG lipase inhibitor JZL184 to increase 2-AG levels also inhibited the development of colitis [173]. Mice treated with JZL184 had less colon alteration and lower expression of pro-inflammatory cytokines, and these effects were abolished by the antagonists AM251 (CB_1_) and AM630 (CB_2_).

Several groups tested the impact of a CB_2_ receptor agonist in the IBD models. The CB_2_-selective agonists JWH-133 and AM1241 both protected against TNBS-induced colitis, whereas AM630 worsened it [197]. The non-psychotropic cannabinoid cannabigerol (CBG) was tested in DNBS-induced colitis and was found to reduce the colon weight/colon length ratio (an indirect marker of inflammation), MPO activity, and iNOS expression by a CB_2_-dependent mechanism [198]. Finally, the plant metabolite and unconventional CB_2_ agonist (*E*)-β-caryophyllene (BCP) was also evaluated in a model of DSS-induced colitis. Oral administration of BCP decreased micro- and macro-scopic colon damage, MPO activity, NF-κB activation, and pro-inflammatory cytokine production [199].

This wide array of CB_2_ receptor agonists being able to improve IBD in animal models prompted the development of highly selective compounds that could be used to treat the disease in humans. In this regard, a research group synthesized a series of CB_2_-selective agonists and tested the resulting lead compounds in models of experimental colitis [200, 201]. Intra-peritoneal injection of the agonists was effective at protecting mice against colitis. Of note, a selective compound that is orally effective in experimental colitis was later synthesized [202].

## Conclusion

In light of the evidence that was generated over the past two decades by the scientific community, we can draw a few general conclusions regarding the role of the CB_2_ receptor. First, it is mainly found in immune tissues and is expressed in most immune cell types. Second, its deletion in animals usually causes an exacerbated inflammatory phenotype in several models, due to an upregulation of immune cell functions. Third, CB_2_ activation by cannabinoids, either in vitro or in vivo, usually decreases inflammatory cell activation. Finally, the administration of CB_2_ agonists in animal models of inflammatory disease can slow the progression of some diseases, in addition to reducing inflammation.

Several questions still need to be investigated. For example, there is no consensus regarding the expression of the CB_2_ receptor in non-immune brain cells, and the role that CB_2_ might play in brain functions is unknown. Moreover, the impact of endocannabinoids on immune cells is still unclear. While most animal studies show that the blockade of endocannabinoid hydrolysis results in less inflammation, it is not possible to tell whether these effects are caused only by CB_2_ activation and whether the opposite would occur in humans. In this respect, endocannabinoids can induce human leukocyte migration (Table 4). However, the impact of endocannabinoid metabolites on leukocyte functions is not well defined, and this should be addressed before endocannabinoid hydrolysis inhibitors that can be considered as a valid strategy to enhance CB_2_ receptor signaling [102]. Finally, the few CB_2_ agonists that are currently being developed aim at treating inflammatory pain [203–205]. Perhaps, these novel compounds are worthy of sparking new studies to define their putative beneficial role in inflammatory diseases.

## Abbreviations

2-AG: 2-Arachidonoyl-glycerol
AA: Arachidonic acid
AEA: *N*-Arachidonoyl-ethanolamide
AM1241: (2-Iodo-5-nitrophenyl)-(1-(1-methylpiperidin-2-ylmethyl)-1H-indol-3-yl)methanone
AM630: 6-Iodo-2-methyl-1-[2-(4-morpholinyl)ethyl]-1H-indol-3-yl](4-methoxyphenyl)methanone
CB65: *N*-Cyclohexyl-7-chloro-1-[2-(4-morpholinyl)ethyl]quinolin-4(1H)-one-3-carboxamide
cAMP: Cyclic adenosine monophosphate
CBD: Cannabidiol
CBG: Cannabigerol
CBN: Cannabinol
COX: Cyclooxygenase
CP 55,940: (–)-*Cis*-3-[2-hydroxy-4-(1,1-dimethylheptyl)phenyl]-*trans*-4-(3-hydroxypropyl)cyclohexanol
Δ^9^-THC: (–)-Δ^9^-Tetrahydrocannabinol
ERK-1/2: Extracellular signal-regulated kinases-1/2
FAAH: Fatty acid amide hydrolase
GFP: Green fluorescent protein
GIRK: G-protein-coupled inwardly rectifying potassium (channel)
GP 1a: *N*-(Piperidin-1-yl)-1-(2,4-dichlorophenyl)-1,4-dihydro-6-methylindeno[1,2-*c*]pyrazole-3-carboxamide
GP 2a: *N*-Cyclohexyl-1-(2,4-dichlorophenyl)-1,4-dihydro-6-methylindeno[1,2-*c*]pyrazole-3-carboxamide
GPCR: G-protein-coupled-receptor
HU-210: 3-(1,1′-Dimethylheptyl)-6aR,7,10,10aR-tetrahydro-1-hydroxy-6,6-dimethyl-6H-dibenzo[b,d]pyran-9-methanol
HU-308: 4-[4-(1,1-Dimethylheptyl)-2,6-dimethoxyphenyl]-6,6-dimethylbicyclo[3.1.1]hept-2-ene-2-methanol
IP_3_: Inositol 1,4,5-trisphosphate
JTE 907: *N*-(1,3-Benzodioxol-5-ylmethyl)-1,2-dihydro-7-methoxy-2-oxo-8-(pentyloxy)-3-quinolinecarboxamide
JWH 015: (2-Methyl-1-propyl-1*H*-indol-3-yl)-1-naphthalenyl-methanone
JWH 133: (6a*R*,10a*R*)-3-(1,1-Dimethylbutyl)-6a,7,10,10a-tetrahydro-6,6,9-trimethyl-6*H*-dibenzo[*b*,*d*]pyran
L-759,633: (6a*R*,10a*R*)-3-(1,1-Dimethylheptyl)-6a,7,10,10a-tetrahydro-1-methoxy-6,6,9-trimethyl-6*H*-dibenzo[*b*,*d*]pyran
L-759,656: (6a*R*,10a*R*)-3-(1,1-Dimethylheptyl)-6a,7,8,9,10,10a-hexahydro-1-methoxy-6,6-dimethyl-9-methylene-6*H*-dibenzo[*b*,*d*]pyran
LOX: Lipoxygenase
MAG: Monoacylglycerol
MAPK: Mitogen-activated protein kinases
NADA: *N*-Arachidonoyl-dopamine
PI3K: Phosphoinositide 3-kinase
PKC: Protein kinase C
PLC: Phospholipase C
PTX: Pertussis toxin
SER 601: *N*-(Adamant-1-yl)-6-isopropyl-4-oxo-1-pentyl-1,4-dihydroquinoline-3-carboxamide
WIN 55,212-2: [(3R)-2,3-Dihydro-5-methyl-3-(4-morpholinylmethyl)pyrrolo[1,2,3-de]-1,4-benzoxazin-6-yl]-1-naphthalenyl-methanone, monomethanesulfonate
SR141716A: *N*-(Piperidin-1-yl)-5-(4-chlorophenyl)-1-(2,4-dichlorophenyl)-4-methyl-1*H*-pyrazole-3-carboxamide hydrochloride
SR144528: 5-(4-Chloro-3-methylphenyl)-1-[(4-methylphenyl)methyl]-*N*-[(1S,2S,4R)-1,3,3-trimethylbicyclo[2.2.1]hept-2-yl]-1H-pyrazole-3-carboxamide
